# Intravenous chloral hydrate anesthesia provides appropriate analgesia for surgical interventions in male Sprague-Dawley rats

**DOI:** 10.1371/journal.pone.0286504

**Published:** 2023-06-23

**Authors:** Rachel Ward-Flanagan, Clayton T. Dickson

**Affiliations:** 1 Neuroscience and Mental Health Institute, University of Alberta, Edmonton, Alberta, Canada; 2 Department of Psychology, University of Alberta, Edmonton, Alberta, Canada; 3 Department of Physiology, University of Alberta, Edmonton, Alberta, Canada; 4 Department of Anaesthesiology & Pain Medicine, University of Alberta, Edmonton, Alberta, Canada; University of Illinois at Urbana-Champaign, UNITED STATES

## Abstract

**Background:**

The use of chloral hydrate as a sole maintenance anesthetic agent in rodent research has been controversial due to statements made in reference literature conflicting with results of primary research studies regarding its analgesic efficacy, and because of its associated tissue damage when administered intraperitoneally.

**Objective:**

Our aim was to assess the analgesic efficacy of chloral hydrate using an intravenous (i.v.) route of administration, in order to prevent the local tissue irritation or ileus that has been previously reported using intraperitoneal (i.p.) routes.

**Methods:**

We measured tail withdrawal latencies to a nociceptive thermal stimulus (infrared beam) in Sprague-Dawley rats–first when awake (unanesthetized), and then subsequently during i.v. chloral hydrate anesthesia. During anesthesia we also measured ongoing heart and respiration rates.

**Results:**

Withdrawal latencies during chloral hydrate anesthesia were significantly higher, and often maximal, indicating a robust analgesic effect. Importantly, both respiration and heart rate remained unchanged following exposure to the nociceptive stimulus, and were comparable to values observed under other anesthetics and during natural sleep.

**Conclusions:**

Together with previous studies, these results demonstrate that i.v. chloral hydrate provides excellent anesthetic depth and analgesic efficacy for surgical manipulations in rats.

## Introduction

For clinical, veterinary, and research applications, general anesthesia is characteristically composed of several discrete endpoints, including: a reversible loss of consciousness, akinesia, amnesia, and analgesia [1]. By this definition, the use of chloral hydrate in animal research has been contraindicated due to assertions it that does not provide adequate analgesia to meet the requirements of a sole anesthetic agent for induction or maintenance [2, 3]. Furthermore, chloral hydrate has other complications related to local tissue irritation and ileus when administered via intraperitoneal (i.p.) injection [4].

Troublingly, the most common claims of insufficient analgesia during chloral hydrate anesthesia appear in reference textbooks where these statements are made without citation to any experimental studies [5, 6]. In direct contrast, our review of the primary neurobiological literature found studies that directly contradicted these claims. For example, comparative studies have demonstrated that the anesthesia and analgesia produced by chloral hydrate is comparable to other commonly used research anesthetics, such as ketamine-xylazine, pentobarbital, and urethane [7, 8]. In addition, the active metabolite of chloral hydrate, 2,2,2-trichloroethanol, has been demonstrated to modulate membrane currents and subsequently inhibit pain transmission in mammalian dorsal root ganglion neurons [9, 10], suggesting a direct means of attenuating pain sensation at the level of the spinal cord itself. Furthermore, when nuclei implicated in the modulation of descending endogenous antinociception (such as the ventrolateral periaqueductal gray, or A5-7 noradrenergic cell groups) are chemically lesioned, analgesia is attenuated (as measured by a decrease in tail flick latency to a thermal nociceptive stimuli) in rats anesthetized with either ketamine or chloral hydrate [11]. Taken together, these studies show that chloral hydrate not only exhibits analgesic effects at the cellular level, but is comparable to other commonly used research anesthetics in terms of supraspinal targets and behavioural effects and as such is an equally appropriate anesthetic for surgical procedures.

However, while other studies have also found that chloral hydrate produced appropriate depth of anesthesia and analgesia for surgical tolerance, there have been valid criticisms due to the post-surgical development of peritonitis and corresponding pathological complications (i.e weight loss and increased stress hormones) when it is administered intraperitoneally [12]. Consequently, our aim was to use a modern and standardized methodology to assess the efficacy of analgesia during intravenous (i.v.) chloral hydrate anesthesia in male Sprague-Dawley rats, and thereby determine whether chloral hydrate is suitable for use as a sole anesthetic agent for maintenance of anesthesia in rodent research. To that end, we measured withdrawal responses in rats to noxious thermal stimuli both while unanesthetized and while anesthetized with chloral hydrate, using an i.v. delivery of chloral hydrate in order to both circumvent the immunological complications associated with the traditional i.p. method and to allow for a more consistent plane of anesthesia. We hypothesized that i.v. chloral hydrate would provide adequate analgesia and increase tail withdrawal latencies to a noxious thermal stimulus, in correspondence with previous studies showing that chloral hydrate provides excellent depth of anesthesia and analgesia.

## Materials & methods

### Subjects

Data were collected from 7 naïve male Sprague-Dawley rats (Charles River; CD 001) weighing on average 319.9 ± 6.0 g (mean ± SEM). Prior to experimentation, rats were kept on a 12-hour light/dark cycle at 20 ± 1⁰C, in polycarbonate shoebox-shaped 42 x 25.5 x 18 cm ventilated cages (Tecniplast, Buguggiate VA, Italy), containing no more than 4 rats per cage. Cages were lined with aspen chip bedding (Living World, Rolf C. Hagen Inc, Baie d’Urfé, QC, Canada) and also contained a plastic tube for enrichment, and standard rat chow (5001, LabDiet, St. Louis, MO) and water (demineralized) were provided *ad libitum*. Welfare checks were performed daily prior to experiments. All methods used in this study conform to the animal use protocol (protocol number: 092) approved by the Biological Sciences Animal Care and Use Committee of the University of Alberta, in accordance with the guidelines established by the Canadian Council on Animal Care. In an effort to reduce our overall number of animals used, a subset of these animals (n = 5) were used in subsequent experiments that are not reported here.

#### Baseline withdrawal assessment

Rats were initially placed in a Hargreaves apparatus (Ugo Basile, Gemonio, VA, Italy) for 15 minutes in order to acclimate to the chamber and reduce movement. Upon acclimation, tail withdrawal latencies to thermal stimulation were recorded by applying an infrared (IR) beam to the approximate middle of the tail at an intensity level of 57 (arbitrary units). The intensity chosen was based on a pilot study with 2 naïve rats, since it produced an average tail withdrawal latency of ~10 seconds [13]. We recorded 5 separate trials of tail withdrawal latency per animal, allowing for recovery for a minimum of one minute between each trial. As the intensity of the IR beam increases during the trial, each trial was limited to a maximum of 30 seconds (3x the average baseline withdrawal latency) to ensure no dermal damage occurred trial to trial [14].

#### Surgery & anesthesia

Following tail withdrawal tests, rats were anesthetized in a plexiglass anesthetic chamber using 4% isoflurane in medical (100%) oxygen. Once the animal exhibited a loss of righting reflexes, it was transferred to a nosecone and maintained at 1.5–2.5% isoflurane and implanted with a flexible silicone jugular catheter (Silastic 508–004, Dow Corning, Midland, MI). Isoflurane was then discontinued and the animal was switched to chloral hydrate anesthesia (Sigma-Aldrich, Oakville, ON; (≥98% MQ200; 0.1 g/mL in phosphate-buffered saline) using an i.v. bolus dose of 200 mg/kg (0.64±0.01 mL based on the previously reported average weight). The rat was then moved to a servo-driven heating pad (TR-100; Fine Sciences Tools, Vancouver, BC) to maintain core body temperature at ~37°C (36.8±0.3°C, n = 7), and placed on a continuous i.v. infusion of chloral hydrate at a rate of 150 mg/kg per hour (0.48±0.01 mL based on the previously reported average weight). We chose this concentration to attenuate the total volume of fluid administered, on average the animals received 1.60±0.03 mL over the course of 2 hours. Animals were then secured in a stereotaxic frame and monitored for any adverse reaction to ensure they were at a surgical plane of anesthesia (Kopf Instruments, Tujunga, CA, USA). If any reaction (i.e paw movement, head shake) was observed, the animal was given an additional small bolus (<0.05 mL/<5 mg) of chloral hydrate until a surgical plane was attained and the animal could be secured within the stereotaxic frame without any reactionary movement. These boluses were recorded with any other pre-trial period boluses, as outlined below.

Heart rate (HR) and respiratory rate (RR) were continuously monitored. HR was measured using a pulse pressure transducer (AD Instruments, Colorado Springs, CO) connected to the left hind paw, while RR was characterized via a thermocouple wire (30 gauge Type K; Thermo Electric Co., Inc.; Brampton, ON, Canada) placed in front of the right nasal passage. Any indication of loss of a surgical plane of anesthesia (increased HR and RR, confirmed with a hindlimb withdrawal to toe-pad pressure) was followed quickly by administration of small (<0.05 mL/<5 mg) bolus increments of chloral hydrate until a clinical plane was restored as defined by a loss of reflexive withdrawal to a hind paw pinch. Small boluses (<0.05 mL/<5 mg) were also administered when the syringe body was refilled to account for the interruption of the continuous infusion. The majority of all boluses for both the maintenance of anesthetic plane and for syringe refills were administered prior to any trials for nociception. The total additional chloral hydrate administered during the pre-trial period was ~12% of the continuous dose per hour (0.18±0.01 mL/18±1 mg, n = 7). Only 2 animals received boluses during the trial period, and only for refill purposes, not due to a loss of surgical plane (0.04±0.01 mL/4±1 mg, n = 2).

#### Anesthetized withdrawal assessment

Rats received a continuous infusion of chloral hydrate for a minimum of 1 hour prior to testing for analgesia, to ensure complete metabolism of any residual isoflurane. Using the same protocol as in the baseline withdrawal assessment, we applied an IR beam at the same intensity level and recorded 5 separate trials of tail withdrawal latency per animal, allowing for recovery for at least one minute between each trial. As before, each trial was limited to a maximum of 30 seconds to ensure no dermal damage occurred trial to trial. Following termination of the experiment, rats were deeply anesthetized using urethane (Sigma-Aldrich; ≥99% MQ200; 1.7 g/kg), as per our locally approved protocol, and then exsanguinated by transcardial perfusion with saline.

#### Data processing & statistical analyses

Thermocouple and transducer signals were amplified at a gain of 1000 and then filtered between 0.1 and 500 Hz using an AC amplifier (Model 1700, A-M Systems Inc.) and sampled at 1000 Hz. All signals were recorded using a PowerLab AD board in conjunction with LabChart Pro (AD Instruments). Average respiration and heart rate were computed from 40-minute baseline recordings taken prior to any IR stimuli trials, and were analyzed using custom scripts for MATLAB (Mathworks; Natick, MA), which processed the signals in spectral time windows 30-seconds in duration, with a frequency resolution of 0.167 Hz.

Due to the complication of movement artefacts in the heart and respiratory signals that often arose during the IR trials from positioning the tail on the IR beam, pair-wise comparisons of pre-IR and IR heart and respiration rate were analyzed for 3 trials per animal where there were no movement artefacts. These physiological analyses were computed by using the peak analysis function in LabChart to assess the instantaneous (cycle-by-cycle) period of heart and respiration rate. The threshold for peak detection was adjusted for pair-wise comparisons to account for any noise in the signal, with an average threshold of 1.84±0.09 standard deviations (SD) from baseline for respiration analyses, and an average threshold of 1.87±0.04 SD from baseline for heart rate analyses. One animal was excluded from respiration analyses due to signal dropout.

Arithmetic means were computed for individual data for each animal within both conditions being compared: baseline unanesthetized and anesthetized for withdrawal latencies, and the 30 seconds pre-IR and during IR for respiration and heart rate during anesthesia. Overall means were then computed for each condition along with SEM. Statistical tests included a Shapiro-Wilk test for normality on both baseline (unanesthetized) and anesthetized, and pre-IR and IR datasets, followed by a paired t-test to compare both conditions within-subjects. These statistical tests were performed using Prism 8 (GraphPad Prism Software Inc, San Diego, CA), using a significance level of α = 0.05. Power analysis was computed post-hoc for latency data and we determined that maximal power was obtained with a minimum group size of 4. Additionally, we determined that the minimum detectable difference for these type of data was 5.18 seconds.

Linear trend analysis as a function of time during exposure to the noxious IR stimuli under chloral hydrate was also performed on both heart and respiration rate to detect any positive-going trends of these signals during the ramping intensity of temperature. These were conducted for individual trials, and for all trials collapsed across animals, separated by trials in which a withdrawal took place, versus those in which no withdrawal was observed. For these aggregate analysis, data were normalized on a trial-wise basis to the individual trial HR or RR average, respectively. These analyses were conducted in Origin (Version 2022 OriginLab Corporation, Northampton, MA, USA).

## Results

During chloral hydrate anesthesia, the average HR was 384±24.0 beats per minute (n = 7), and the average RR was 108±5.40 breaths per minute (n = 7), which fall between values previously reported for urethane anesthetized rats (~444.0 beats per minute HR; ~126 breaths per minute RR) and naturally sleeping rats during slow-wave sleep (~330 beats per minute HR; ~96.0 breaths per minute RR), and as such are consistent with unconsciousness [15, 16].

**Fig 1:**
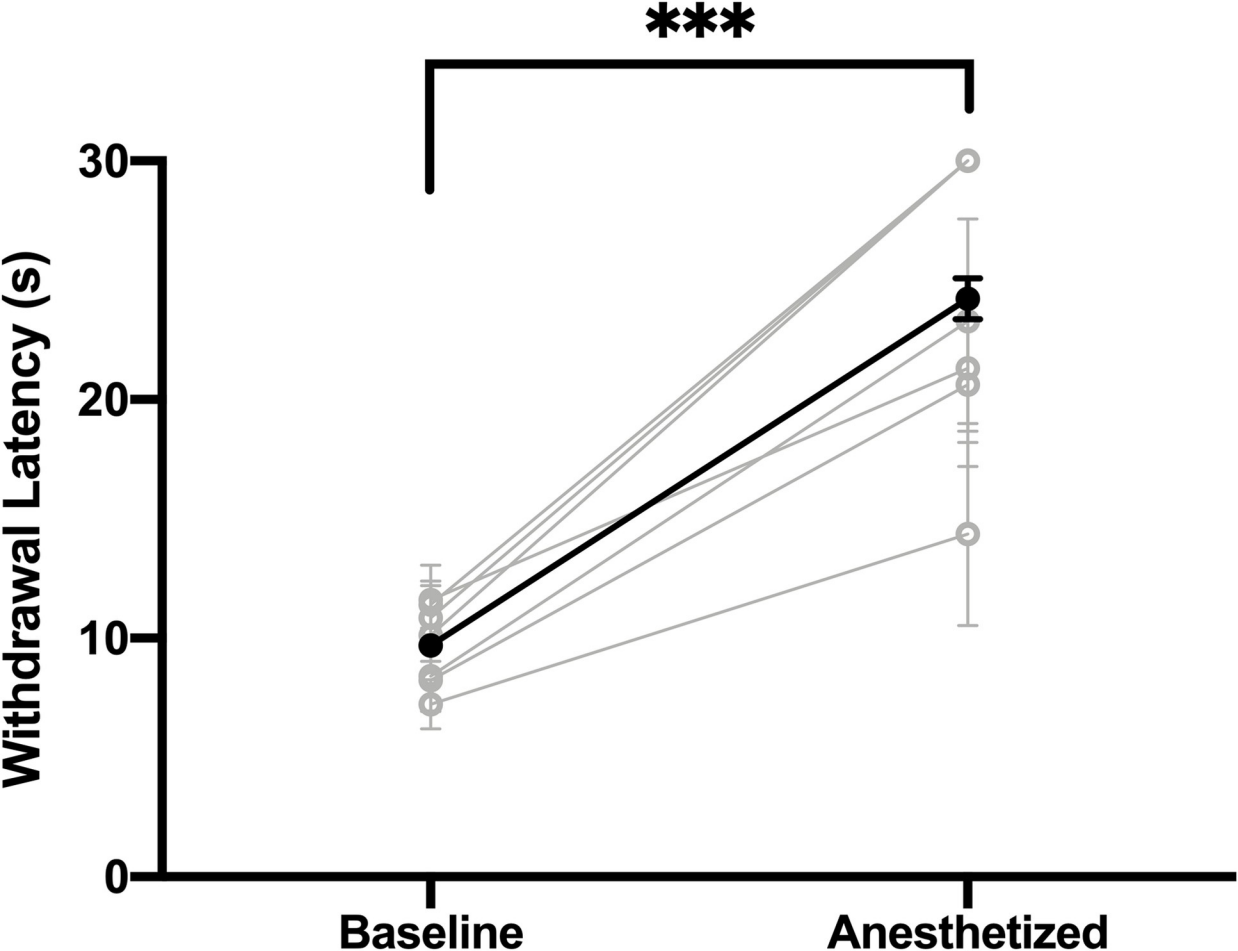
Chloral hydrate anesthesia significantly increases latency of tail withdrawal from a nociceptive stimulus. Within-subject comparison of tail withdrawal latencies to a nociceptive stimulus while unanesthetized and while subsequently anesthetized with chloral hydrate anesthesia (200mg/kg initial bolus; 150 mg/kg per hour continuous intravenous infusion). Withdrawal latencies increased significantly during chloral hydrate anesthesia in every rat tested (individual values represented in grey; average represented in black). ***p<0.0001.

Compared to values during unanesthetized conditions, tail withdrawal latencies were significantly longer under chloral hydrate anesthesia in 6 of the 7 rats tested, as determined by a Student’s t-test for each animal. Furthermore, in 3 of the 7 animals tested, and across 57% of all anesthetized trials, no withdrawal took place within the 30 second limit (Fig 1). A Shapiro-Wilk test showed that both unanesthetized (W(6) = 0.9043, p = 0.3580) and anesthetized (W(6) = 0.8684, p = 0.1798) data sets were normal, and a paired t-test revealed that this increase in withdrawal latency was highly significant (t(6) = 7.711, p = 0.0002).

**Fig 2:**
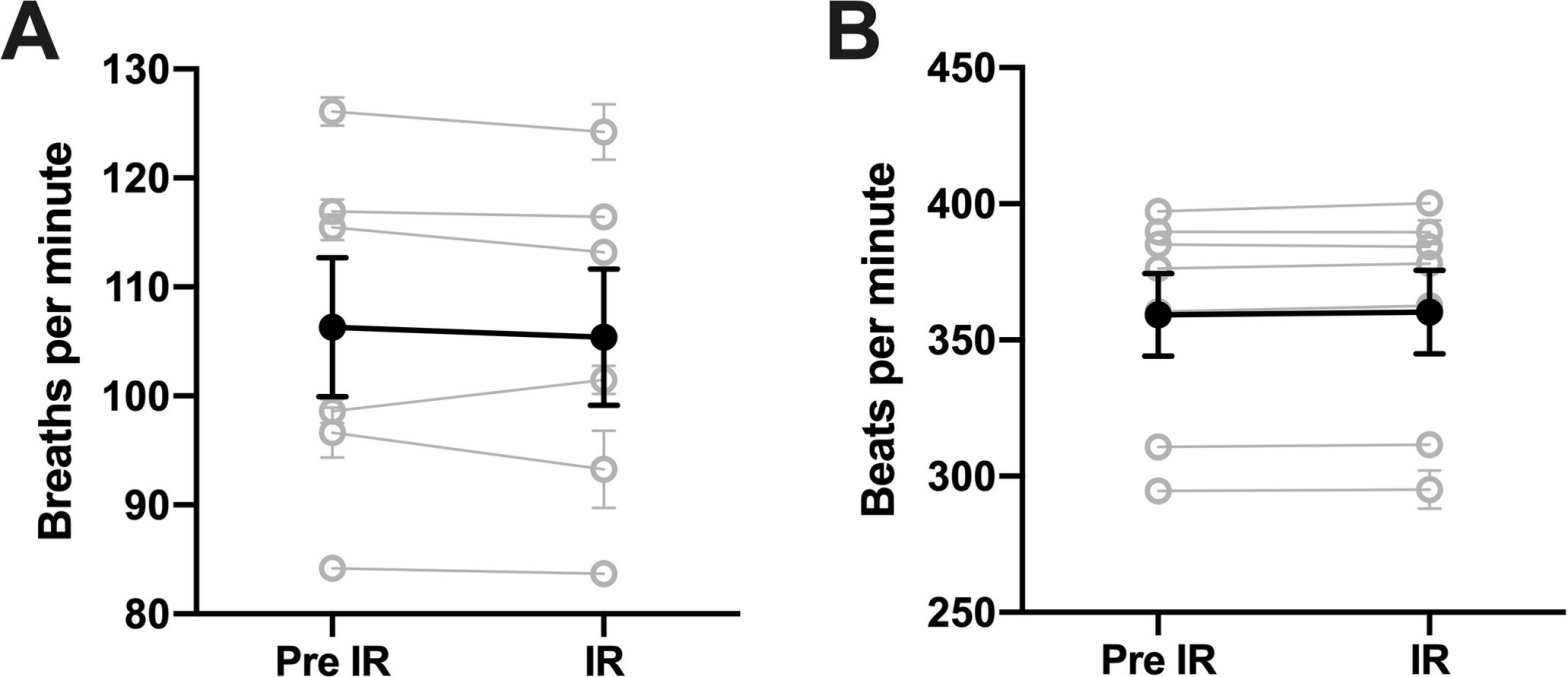
Heart and respiratory rate remain unchanged during nociceptive stimulation under chloral hydrate anesthesia. (A) Within-subject comparison of respiration rate 30 seconds pre-IR stimulus and during IR stimulus averaged over 3 separate trials (individual averages are represented in grey, grand average of all animals is represented in black). (B) Within-subject comparison of heart rate 30 seconds pre-IR stimulus and during IR stimulus averaged over 3 separate trials (individual averages are represented in grey, grand average of all animals is represented in black).

Moreover, there were no changes in either respiration or heart rates in response to the nociceptive thermal IR stimulus (Fig 2). A paired t-test comparison of the 30 seconds immediately preceding an IR stimulus (pre-IR) to the 30 second period during which the IR beam was delivered yielded no significant differences in the RR (t(5) = 1.070, p = 0.334) following a Shapiro-Wilk test for normality which showed that both unanesthetized (W(5) = 0.9467, p = 0.7137) and anesthetized (W(5) = 0.9642, p = 0.8517) data sets were normal. Similarly, in measuring HR, both unanesthetized (W(6) = 0.8472, p = 0.1158) and anesthetized (W(6) = 0.8527, p = 0.1302) data sets were normal and no significant changes (t(6) = 2.061, p = 0.085) were observed when comparing the 30s pre-IR to IR.

**Fig 3:**
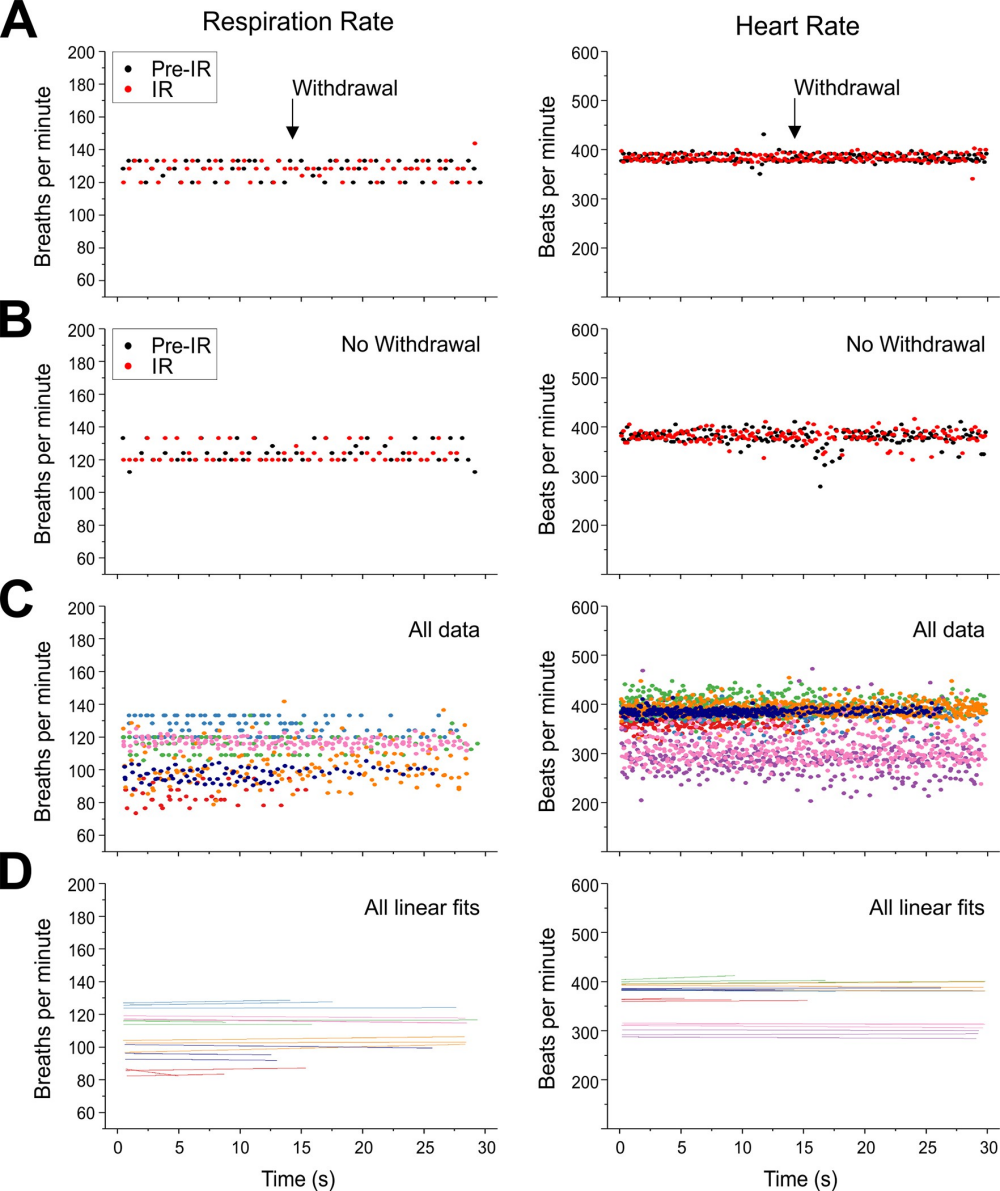
Heart and respiratory rate remain unchanged with or without tail flick response during nociceptive stimulation under chloral hydrate anesthesia. (A) Within-subject comparison of instantaneous (cycle-by-cycle) respiration rate (left) and heart rate (right) 30 seconds pre-IR stimulus (in black) and during IR stimulus (in red) for a single trial. The ramped thermal nociceptive stimulation was terminated at the arrowhead (14.5 s) due to a tail flick reaction. (B) Within-subject comparison of instantaneous (cycle-by-cycle) respiration rate (left) and heart rate (right) 30 seconds pre-IR stimulus (in black) and during IR stimulus (in red) for a single trial. The ramped thermal nociceptive stimulation was continued throughout the 30 s trial since no withdrawal occurred (C) Instantaneous respiration rate (left) and heart rate (right) during IR exposure for all trials (3 trials per animal). Each animal is denoted by a separate colour. (D) Linear fits of the instantaneous respiration rate (left) and heart rate (right) for individual trials during IR exposure, with each animal denoted by a separate colour, as in C. In no case was there a significant positive linear fit.

To more stringently evaluate potential changes in RR and HR in response to the ramping noxious thermal stimulus, we evaluated if any positive trends (i.e increases in RR or HR) were evident during IR exposure. Examples of the cycle-by-cycle analysis for both respiratory and heart rate signals as a function of time during these trials is shown in Fig 3A–3C. Consistent with our previous analysis above, when the relative distribution of the instantaneous (cycle-by-cycle) HR and RR of the 30s pre-IR was compared to the 30s during in which IR exposure occurred (overlaid in red), no differences were observed, irrespective of whether withdrawal occurred Fig 3A, 3B–note: panels A and B are two different trials from the same animal). When we visualized the instantaneous HR and RR during exposure to the IR (i.e cutting off measures at any tail withdrawal) for all trials, we observed a wide distribution of average HR and RR across animals (Fig 3C). Therefore, we assessed the linear fit for individual trials (Fig 3D) which revealed no significant positive trends for either respiration or heart rate cycles across IR applications. Furthermore, trial-normalized data collapsed across all animals showed no significant positive trends for either HR or RR, regardless of whether a withdrawal occurred or not (RR withdrawal trials: F(1,236) = 0.05, p = 0.82, r = -0.01; RR non-withdrawal trials, F(1,417) = 0.07, p = 0.79, r = 0.01; HR withdrawal trials, F(1,874) = 0.05, p = 0.71, r = 0.01; HR non-withdrawal trials, F(1,1820) = 0.08, p = 0.78, r < -0.01). Thus, there were no systematic increases in either RR or HR as in response to the increasing noxious thermal stimulus over time.

## Discussion

Our data demonstrate that there is a significant increase in the latency of withdrawal responses, together with no changes in ongoing physiological responses such as respiration or heart rate in response to a nociceptive thermal stimulus under continuous i.v. chloral hydrate anesthesia. These results are consistent with prior reports from the primary literature that chloral hydrate provides excellent anesthetic depth and analgesia for surgical manipulations in rats [7, 8].

While the assessment of nociception using reflexive responses can be a difficult task due to the many inter-related physiological factors that can influence behavioural outcomes [14], we endeavoured to mitigate these confounds by using a within-subjects design to compare the naïve unanesthetized baseline to the anesthetized state, on the same day, at the same IR intensity across all subjects, and at the same location on the tail. Though a relatively simple protocol, our approach controls for much of the variance that can often affect thermal nociceptive measures (i.e ambient room temperature, stimulation site, and intensity, etc.) [14, 17]. Our within-subjects design also informed our choice to analyze our data as the change of raw latency values from baseline for each animal, rather than as a percentage of the maximum latency, which would be dependent on our arbitrary cut-off, and which would lead to the statistical fallacy of comparing percentages of percentages [14]. Thus, we are confident that our data is a fair representation of the analgesic effect of chloral hydrate on the tail flick response to a nociceptive stimulus in rats.

The occurrence of tail withdrawals under chloral hydrate in our results are a further indication that the effect observed is due to analgesia, rather than an artefact of immobility or paralysis which might cause an inability to move despite the animal still perceiving pain. Furthermore, while the tail flick withdrawal is typically considered a spinal reflex, lower intensity stimuli which produce a delayed baseline withdrawal (>5s) are generally thought to engage higher-order supraspinal structures in order to process pain and execute the withdrawal behaviour [14, 17, 18]. This line of evidence, when taken together with the study showing that chemical lesions to the vlPAG or descending noradrenergic neurons (both of which are thought to modulate endogenous antinociception) significantly decreases tail flick latency under chloral hydrate anesthesia [11], suggests that the analgesic effect we observed is mediated via supraspinal structures.

Our monitoring of other physiological variables, specifically respiration and heart rate further support our conclusion that chloral hydrate provides adequate analgesia. Decreases in RR and HR are indicators of unconsciousness that also apply to sleep [19]. Consequently, it is typically required in both clinical and veterinary surgical protocols to monitor respiration and heart rate, as increases in either are indicative of a decrease in anesthetic depth, and often precede an imminent return of consciousness [5, 20]. Indeed, it has been shown that in male Sprague-Dawley rats receiving a sub-anesthetic dose of halothane (0.75%) that HR increases in response to a similar noxious thermal stimulus (as used in the present study) occurred in conjunction with a tail flick response [21]. Given that both RR and HR are centrally controlled/modulated, this further implies in our studies that there is a lack of centrally processed pain responses–a fundamental assessment of analgesia.

Yet, we acknowledge that our study has limitations. Though we have shown that i.v. administration of chloral hydrate provides an appropriate depth of anesthesia and analgesia necessary to achieve a surgical plane, further experiments will be required to determine if i.v. administration alleviates the post-surgical physiological side effects associated with i.p. routes [12]. In addition, the scope of nociceptive stimuli in our study is limited to a noxious thermal stimulus. However, when considered in the context of previous primary literature, our data adds to and is consistent with reports that the anesthesia and analgesia provided by chloral hydrate is appropriate for surgical manipulations [7, 8, 12, 22], and thus are in stark contrast to widespread claims that chloral hydrate should only be considered as a hypnotic with poor analgesic properties [3, 5, 6].

We stress that our argument is not that analgesics should never be used in conjunction with chloral hydrate anesthesia, but that chloral hydrate provides sufficient analgesia for experimental paradigms which require a surgical plane of anesthesia and aim to minimize confounding factors that multiple drugs can introduce. Although chloral hydrate has been under-utilized mainly due to the erroneous assertion of inadequate analgesia, it appears that when delivered intravenously in rats, it meets the requirements of a sole anesthetic agent for maintenance of anesthesia and should be considered more often by researchers when using the protocol that we have outlined here.
